# Effect of Cellular Location of Human Carboxylesterase 2 on CPT-11 Hydrolysis and Anticancer Activity

**DOI:** 10.1371/journal.pone.0141088

**Published:** 2015-10-28

**Authors:** Yuan-Ting Hsieh, Hsuan-Pei Lin, Bing-Mae Chen, Ping-Ting Huang, Steve R. Roffler

**Affiliations:** 1 Institute of Microbiology and Immunology, National Yang-Ming University, Taipei, Taiwan; 2 Institute of Biomedical Sciences, Academia Sinica, Taipei, Taiwan; Okayama University, JAPAN

## Abstract

CPT-11 is an anticancer prodrug that is clinically used for the treatment of metastatic colorectal cancer. Hydrolysis of CPT-11 by human carboxylesterase 2 (CE2) generates SN-38, a topoisomerase I inhibitor that is the active anti-tumor agent. Expression of CE2 in cancer cells is under investigation for the tumor-localized activation of CPT-11. CE2 is normally expressed in the endoplasmic reticulum of cells but can be engineered to direct expression of active enzyme on the plasma membrane or as a secreted form. Although previous studies have investigated different locations of CE2 expression in cancer cells, it remains unclear if CE2 cellular location affects CPT-11 anticancer activity. In the present study, we directly compared the influence of CE2 cellular location on substrate hydrolysis and CPT-11 cytotoxicity. We linked expression of CE2 and enhanced green fluorescence protein (eGFP) via a foot-and-mouth disease virus 2A (F2A) peptide to facilitate fluorescence-activated cell sorting to achieve similar expression levels of ER-located, secreted or membrane-anchored CE2. Soluble CE2 was detected in the medium of cells that expressed secreted and membrane-anchored CE2, but not in cells that expressed ER-retained CE2. Cancer cells that expressed all three forms of CE2 were more sensitive to CPT-11 as compared to unmodified cancer cells, but the membrane-anchored and ER-retained forms of CE2 were consistently more effective than secreted CE2. We conclude that expression of CE2 in the ER or on the membrane of cancer cells is suitable for enhancing CPT-11 anticancer activity.

## Introduction

CPT-11 (irinotecan) is a clinically important prodrug that is activated to SN-38 *in vivo*. SN-38, a potent topoisomerase I inhibitor, is thought to play the major role in the anti-tumor activity of CPT-11 [1]. CPT-11 displays anticancer activity against a variety of solid tumors, including colorectal cancer, non-small cell and small cell lung cancers, gynecologic cancers, and refractory cervical cancer [2–4]. CPT-11 is approved for first-line therapy in combination with 5-FU/LV for the treatment of metastatic colorectal cancer [5].

Mammalian carboxylesterases (CEs) play important roles in the detoxification and metabolic activation of various drugs and environmental toxicants that contain ester or amide bonds. CEs are a ubiquitously expressed class of enzymes that have been found in different mammalian species such as rabbits [6], rats [7] and human [8, 9]. CE enzymes are mainly localized in the endoplasmic reticulum of cells. Human carboxylesterase 2 (CE2) is believed to be important for the conversion of CPT-11 to SN-38 in humans [10]. CE2, which is mainly distributed in the small intestine, liver and colon, possesses about 100-fold and 2000-fold higher catalytic activity (kcat/ Km) for CPT-11 hydrolysis to SN-38 than CE1 and CE3, respectively [11]. Incubation of human lung adenocarcinoma and melanoma cells with CE2 resulted in higher sensitivity to CPT-11 as compared with cells incubated with CE1 [12] and knockdown of CE2 attenuated CPT-11 cytotoxicity [13]. In addition, clinical data indicate that CPT-11 is not effective against lymphomas and gallbladder tumors that do not express CE2 [14].

Several studies have demonstrated that the expression of CE intracellularly [10, 15–18] or extracellularly [18–23] can increase the sensitivity of cancer cells to CPT-11. Expression of CE2 in the natural ER location may provide an optimal environment for enzyme activity whereas expression of CE2 on the plasma membrane or as a secreted form may enhance accessibility to CPT-11. However, it is difficult to directly compare previous studies due to differences in enzyme expression levels, cell lines and experimental conditions. Here, we directly compared enhancement of cancer cell sensitivity to CPT-11 by CE2 expressed in the ER (erCE2), on the cell membrane (mCE2) or as a secreted form (sCE2). Expression levels were carefully adjusted by sorting cells for similar expression of a F2A-linked green fluorescence protein. We find that mCE2 and erCE2 significantly enhanced cancer cell sensitivity to CPT-11 as compared to secreted CE2.

## Materials and Methods

### Reagents

Biotin-conjugated goat anti-HA IgG was from Vector laboratories (Burlingame, CA). Streptavidin-HRP, Alexa-488 conjugated streptavidin, and rhodamine-conjugated streptavidin were from Jackson ImmunoResearch Laboratories (West Grove, PA). DMEM, mouse anti-beta-actin antibody, sodium bicarbonate and p-nitrophenyl acetate (NPA) were from Sigma-Aldrich (St. Louis, MO). 4',6-diamidino-2-phenylindole (DAPI) and RPMI-1640 were from Invitrogen (Carlsbad, CA). Bovine calf serum (BCS) was from HyClone (Logan, Utah). ^3^H-thymidine was from PerkinElmer (Boston, MA).

### Cells

HCT116 (ATCC No.CCL-247) human colorectal carcinoma cells and BALB/3T3 (ATCC No. CCL-163) mouse fibroblasts were from the American Type Culture Collection (Manassas, VA). EJ human bladder carcinoma cells were a gift from Dr. Konan Peck (Academia Sinica, Taiwan) [24]. GP2-293 human embryonic kidney cells (Clontech, CA, USA) were provided by Dr. Andre Lieber (University of Washington, Seattle, WA, USA). BALB/3T3 cells were cultured in DMEM medium containing 2.98 mg/ml HEPES, 1 mg/ml sodium bicarbonate, and 10% bovine calf serum. EJ and HCT116 cells were cultured in RPMI-1640 medium containing 2.98 mg/ml HEPES, 1 mg/ml sodium bicarbonate, and 10% bovine calf serum. GP293v cells were cultured in DMEM medium containing 2.98 mg/ml HEPES, 1 mg/ml sodium bicarbonate, and 10% fetal bovine serum. Cells were cultured in an atmosphere of 5% CO_2_ in air at 37°C.

### Plasmid constructs

**Fig 1:**
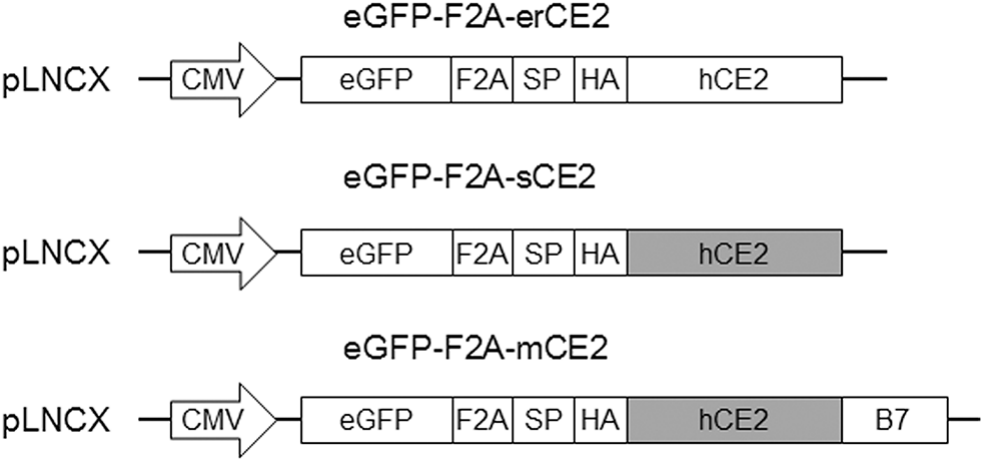
Schematic of CE2 expression constructs. All construct are based on the pLNCX backbone with gene expression under the control of the CMV promoter. The eGFP gene is followed by a F2A sequence and different forms of CE2. HA: hemaglutinin tag, CE2: human carboxylesterase 2, white box: CE2 with ER-retention signal. Gray box: CE2 without ER-retention signal, B7: juxtamembrane Ig-like extracellular, transmembrane domain, and cytoplasmic tail of mouse B7.

The plasmid constructs used for eGFP and CE2 coexpression are shown in Fig 1. We utilized a F2A sequence to link the expression of eGFP and CE2 in the cells. The eGFP gene was placed in front of the F2A sequence and CE2 genes, amplified from cDNA obtained from EJ cells by using specific primers. The HTEL motif at the C-terminus of CE2 was maintained in pLNCX-eGFP-HA-erCE2 to allow retention of CE2 in the ER. The HTEL sequence was truncated from CE2 in pLNCX-eGFP-HA-sCE2 to allow secretion of CE2. To express CE2 on the plasma membrane, the sCE2 gene was fused to cDNA coding for the juxtamembrane Ig-like extracellular, transmembrane domain and cytoplasmic tail of murine B7-1 [25]. The fusion gene was subcloned into pLNCX-eGFP-HA-sCE2 to create pLNCX-eGFP-HA-mCE2. All transgenes were cloned in the retroviral pLNCX (Clontech, Mountain View, CA) backbone with expression under control of the CMV promoter.

### Expression of different forms of CE2

2.5 μg pLNCX-eGFP-HA-mCE2, pLNCX-eGFP-HA-sCE2 or pLNCX-eGFP-HA-erCE2 were cotransfected with 2.5 μg pVSVG (Clontech, Mountain View, CA) into GP2-293 cells by the calcium phosphate method to produce pseudotyped recombinant retrovirus particles allowing expression of membrane-anchored, secreted, or ER-retained CE2. 10^5^ BALB/3T3, EJ or HCT116 cells were infected with retroviral particles and selected with G418 (0.5 mg/ml for 3T3 cells, 0.4 mg/ml for EJ cells and 0.8 mg/ml for HCT116 cells) to create stable cell lines as previously described [26]. Cells were sorted for similar eGFP fluorescence intensity on a fluorescence-activated cell sorter (FACSAria) (BD Biosciences, San Jose, CA) to collect cells with similar CE2 expression.

### Flow cytometry

To detect mCE2 surface expression, 10^6^ 3T3, 3T3-mCE2, EJ, EJ-mCE2, HCT116 and HCT116-mCE2 cells were stained with 10 μg/ml biotin-conjugated goat anti-HA antibody in 50 μl 1% BSA in PBS on ice for 30 min. After washing with cold 1% BSA in PBS twice, the cells were incubated with 18 μg/ml Alexa-488 conjugated streptavidin in 50 μl 1% BSA in PBS on ice for 30 min. The cells were washed with cold 1% BSA in PBS twice. Afterwards, the cells were stained with propidium iodide (Sigma-Aldrich) (1 μg/ml in PBS), and the surface immunofluorescence of 10,000 viable cells was measured with a FACScaliber flow cytometer (Becton Dickinson, Mountain View, CA), and fluorescence intensities were analyzed with Flowjo V3.2 (Tree Star Inc., San Carlos, CA).

### Relationship between eGFP fluorescence and CE2 expression

To verify the relationship between eGFP fluorescence and the expression of membrane-anchored CE2, 3T3-mCE2, EJ-mCE2 and HCT116-mCE2 cells were sorted on a FACScaliber flow cytometer into different populations based on eGFP fluorescence intensity. The expression of mCE2 on the cell populations was then measured on a FACScaliber flow cytometer by immunofluorescence staining of the HA epitope tag present on mCE2. The mean fluorescence intensity of mCE2 was plotted against the eGFP mean fluorescence intensity and least squares regression was used to determine the correlation coefficient.

HCT116-sCE2 and HCT116-erCE2 cells were also sorted to generate cell populations with different eGFP fluorescence intensities. 10^5^ of each population of HCT116-sCE2 and HCT116-erCE2 cells were seeded into 24-well plates for 48 h. Half of the cells were collected and eGFP intensity was measured on a FACScaliber flow cytometer. The relative amounts of soluble CE2 in the culture medium from HCT116-sCE2 cell populations and the relative amounts of intracellular CE2 in cell lysates prepared from HCT116-erCE2 cell populations was determined by immunoblotting with an anti-HA epitope tag antibody as described below. Beta-actin in cell lysates was also immunoblotted as a cell loading control. Relative sCE2 expression was determined by comparing sCE2 band intensities whereas the relative erCE2 expression was calculated as the ratio of erCE2 band intensity divided by actin band intensity. Least squares regression was used to determine the correlations between eGFP fluorescence intensities and relative sCE2 or erCE2 expression.

### Cell proliferation assay

10^5^ parental, mCE2, sCE2 and erCE2 expressing cells were seeded into 6-well cell culture plates (Corning Incorporated, NY) and 10-cm cell culture dishes, respectively. Cells were cultured at 37°C. On days 1 and 2, cells were trypsinized from 6-well cell culture plates. The cells were mixed with trypan blue and the live cells were counted on a Countess automated cell counter (Invitrogen). On days 3 and 4, cells were trypsinized from 10-cm cell culture dishes and the cell number was counted.

### Western blotting

5 x 10^5^ parental, mCE2, sCE2 and erCE2 expressing cells were seeded into 6-well plates overnight. The medium was removed and 2 ml DMEM containing 2% BCS was added. The medium was harvested after 48 h and further centrifuged at 90,000 g for 30 min to obtain cell-free supernatant. The cells were detached with versene, washed twice with PBS and once with lysis buffer (10 mM HEPES-NaOH, pH 7.9, 1.5 mM MgCl_2_, and 10 mM KCl). The cells were suspended in 0.5 ml lysis buffer on ice for 30 min and then broken with a dounce homogenizer. 20 μl cell lysates or culture medium were electrophoresed in a 10% SDS-PAGE, electrotransferred to a PVDF membrane and immunoblotted with biotin-conjugated goat anti-HA IgG (to bind to the HA epitope tag on the N-terminus of CE2) and streptavidin-HRP. The membranes were also blotted with a mouse anti-beta-actin antibody as a cell loading control.

### CE activity assay

We measured the CE activity by following Ross *et al* [27]. 20 μl cell lysate or culture medium were mixed with 130 μl reaction buffer (Tri-HCl, pH 7.4) and 150 μl p-nitrophenyl acetate (pNPA) (500 μM in reaction buffer) and incubated at 37°C. The p-nitrophenol formation was periodically measured at a wavelength of 405 nm during 10 min on a Thermo max microplate reader (Molecular Devices, Sunnyvale, CA). The relative total CE2 activity was calculated as: Relative total activity = nmol p-nitrophenol formation/min/actin amount detected by western blotting.

### Immunofluorescence staining

3 x 10^5^ EJ, EJ-mCE2, EJ-sCE2 and EJ-erCE2 cells were seeded overnight on glass coverslips. The cells were fixed with 2% paraformaldehyde in PBS and then maintained in PBS or incubated with 0.1% Triton X-100 in PBS to permeabilize the cells to allow intracellular staining. These cells were blocked with 1% BSA in PBS and then stained with biotin-conjugated goat anti-HA IgG followed by rhodamine-conjugated streptavidin. Nuclei were stained with DAPI. The CE2 distribution was imaged on an aLSM-700 confocal microscope (Zeiss, Thornwood, NY).

### ^3^H-thymidine incorporation assay

5000 EJ or HCT116 CE2-expressing cells per well were seeded in 96-well culture plates overnight. Graded concentrations of CPT-11 or SN-38 were added into the wells and incubated at 37°C for 48 h. After discarding the supernatant and washed the cells with PBS twice, fresh growth medium containing ^3^H-thymidine (1 μCi/well) was added for another 16 h. The radiation in each well was measured on a Top Count scintillation counter. The results are expressed as % inhibition = c.p.m._(D)_ / c.p.m._(C)_ x 100% where c.p.m. represents counts per minute of drug-treated cells (D) or untreated control cells (C).

### Statistical significance

Statistical significance of differences between mean values was estimated with Excel (Microsoft, Redmond, WA, USA) using the independent t-test for equal variances. P-values of < 0.05 were considered statistically significant.

## Results

### eGFP intensity is proportional to CE2 expression

**Fig 2:**
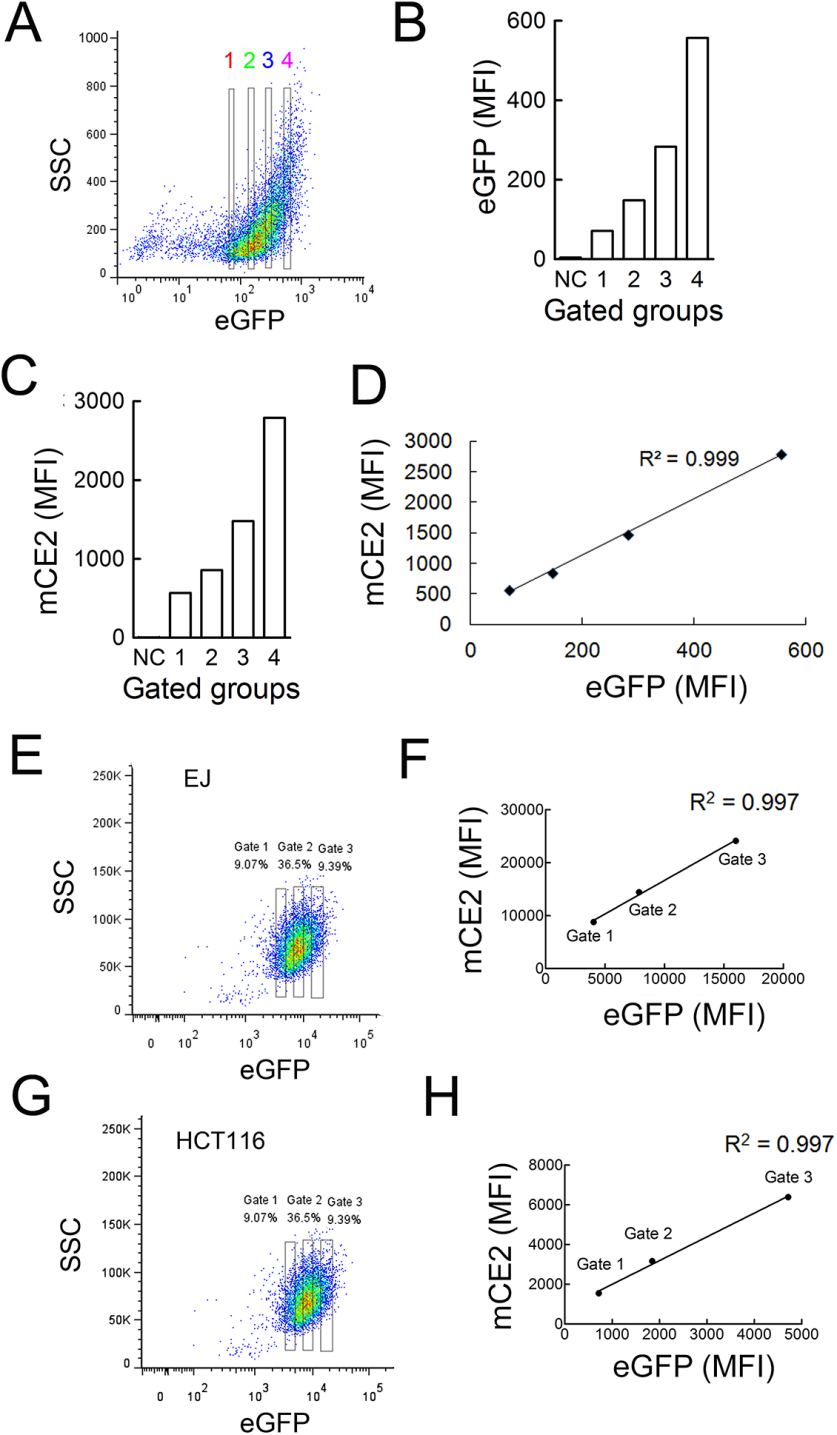
Correlation between eGFP and mCE2 expression. (A) 3T3-mCE2 cells were gated into four groups based on their eGFP fluorescence intensity. The mean fluorescence intensity of eGFP (B) and surface CE2 (C) in the four groups of cells is shown. Surface CE2 expression of 3T3-mCE2 cells was measured by staining with anti-HA antibody followed by Alex 647-conjugated anti-goat IgG antibody. NC represents 3T3 cells stained with the same antibody combination to serve as a negative control. (D) The linear correlation between the eGFP and CE2 mean fluorescence intensity is shown. (E) EJ-mCE2 cells and (G) HCT116-mCE2 cells were gated into three groups based on their eGFP fluorescence intensity. The linear correlation between the eGFP and mCE2 mean fluorescence intensity in (F) EJ-mCE2 cells and (H) HCT116-mCE2 cells are shown.

Comparison of the effects of CE2 location on CPT-11 anti-tumor activity is predicated on expressing similar levels of CE2 in target cells. However, it is difficult to compare CE2 protein levels, especially for the secreted enzyme. To overcome this problem, we used eGFP as a reporter gene to monitor the expression of CE2. The eGFP and various CE2 genes were linked with a F2A sequence, which promotes ribosomal skipping so that one open reading frame can be translated into two proteins [28, 29]. Proteins flanking the F2A peptide theoretically have a high degree of coordinate expression [30]. To investigate if eGFP fluorescence intensity correlated with CE2 expression, BALB/3T3 cells that stably expressed mCE2 (3T3-mCE2 cells) were first generated. The cells were sorted by FACS into four different populations based on their eGFP fluorescence intensity (Fig 2A). The cells were also stained with anti-HA antibody to measure the levels of CE2 on their surface. The eGFP intensity (Fig 2B) and mCE2 expression levels (Fig 2C) from these populations were counted. eGFP fluorescence was highly correlated with mCE2 expression in 3T3-mCE2 cells (Fig 2D).

HCT116 human colon cancer cells are a commonly used cell line for CPT-11 therapy research. We also used EJ human bladder cancer cells for SN-38 cytotoxicity assay in our previous studies [31, 32]. Therefore, EJ cells served as an internal control for cytotoxicity assays. Both cells express low levels of endogenous, intracellular CE2. EJ human bladder cancer and HCT116 human colon cancer cells were infected with recombinant retroviruses to generate stable cell populations that express membrane-anchored CE2 (mCE2), secreted CE2 (sCE2) or endoplasmic reticulum-retained CE2 (erCE2) cells.

**Fig 3:**
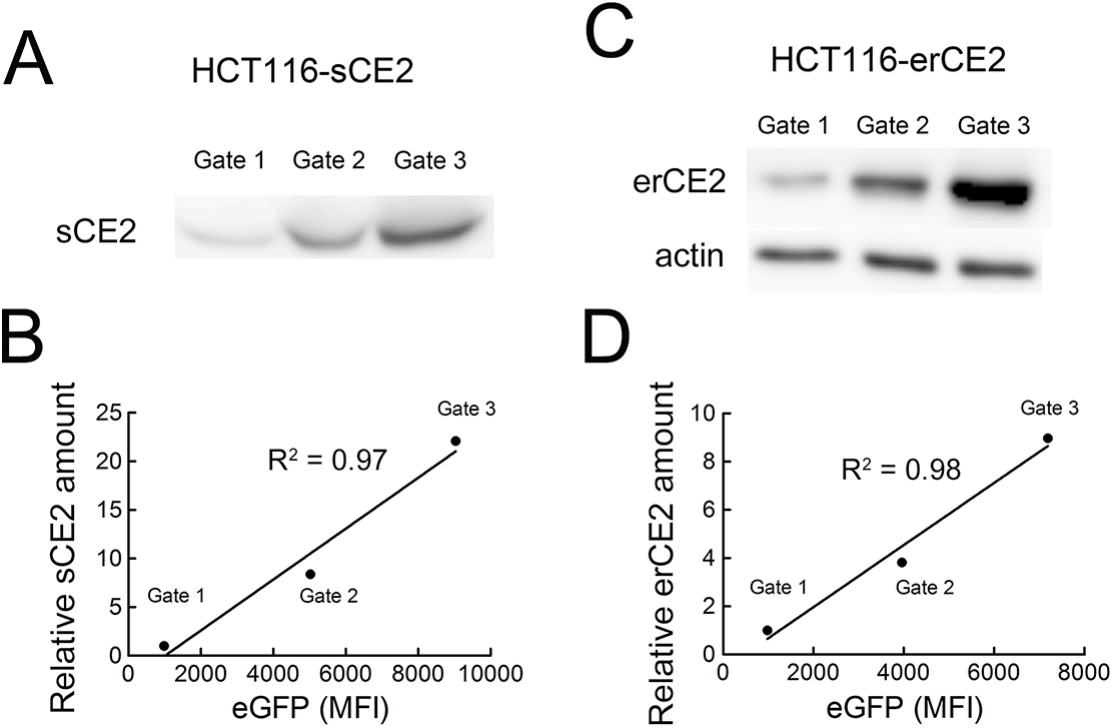
Correlation between eGFP and soluble or intracellular CE2 expression. HCT116-sCE2 and HCT116-erCE2 cells were sorted into populations displaying different eGFP fluorescence intensities. The amount of sCE2 in the culture medium of HCT116-sCE2 cell populations (A) or erCE2 in cell lysates prepared from HCT116-erCE2 cell populations (C) was determined by immunoblotting. The linear correlation between the eGFP fluorescence and the relative amounts of sCE2 (B) or erCE2 (D) are shown.

To determine if eGFP fluorescence correlated with membrane-anchored CE2 on EJ-mCE2 and HCT16-mCE2 cells, FACS was used to collect cells populations that displayed different eGFP fluorescence intensities (Fig 2E and 2G). Immunofluorescence staining of these cell populations with an anti-HA epitope tag antibody to measure mCE2 levels revealed good linear correlations between eGFP fluorescence intensity and mCE2 levels in EJ-mCE2 and HCT16-mCE2 cells (Fig 2F and 2H). We also sorted HCT-116-sCE2 and HCT116-erCE2 cells, which respectively express secreted or intracellular CE2, into different populations based on eGFP fluorescence intensities. The amount of CE2 in each cell population was determined by immunoblotting the culture medium (Fig 3A) or cell lysates (Fig 3C) prepared from identical numbers of HCT-116-sCE2 and HCT116-erCE2 cell populations, respectively. Good correlations were observed between eGFP fluorescence intensities and sCE2 (Fig 3B) or erCE2 (Fig 3D) levels. We conclude that eGFP fluorescence can be used to estimate the relative expression levels of CE2 in cells.

### Characterization of CE2-expressing cells

**Fig 4:**
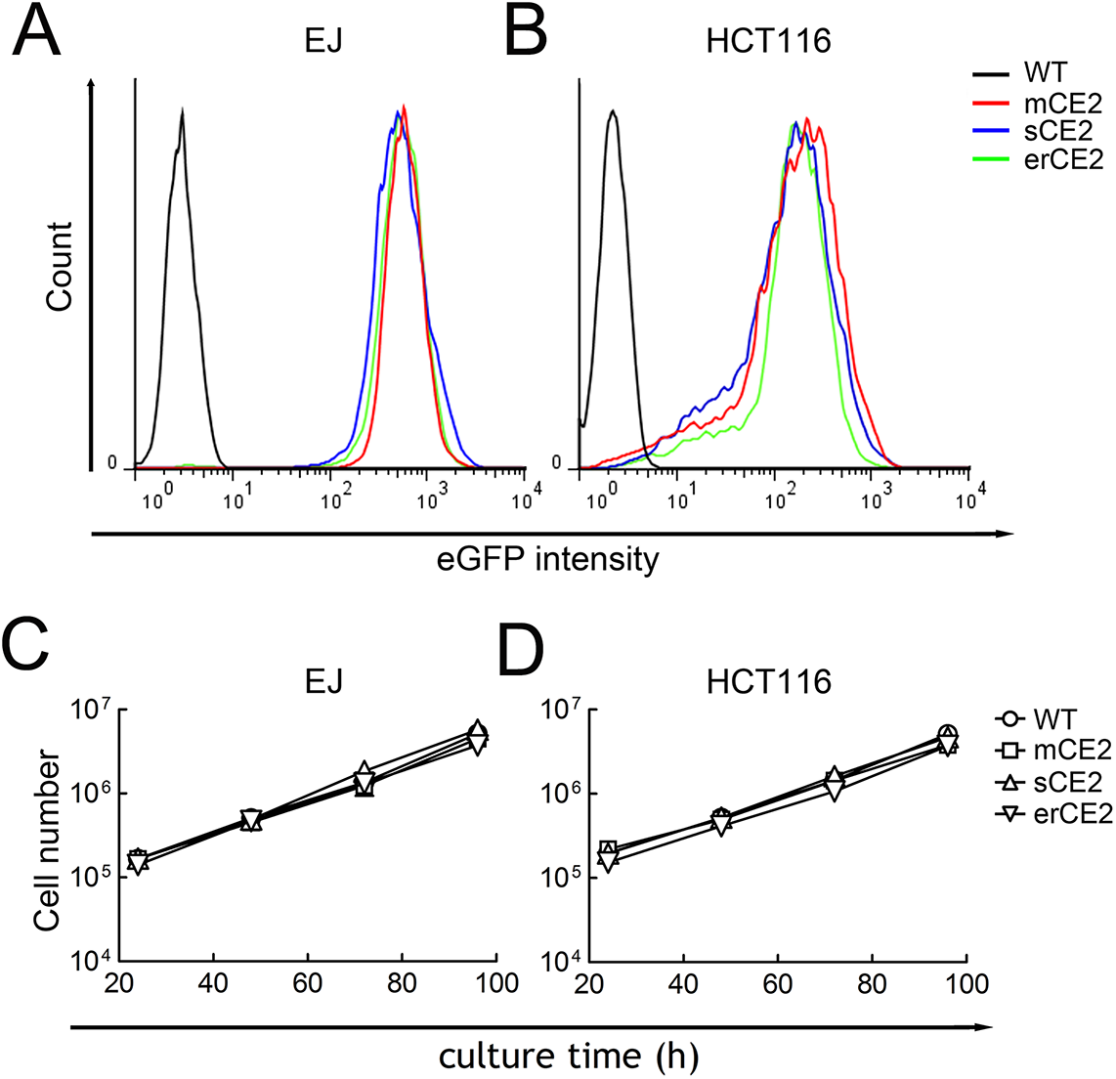
Characterization of EJ and HCT116 cells expressing CE2 in specific cellular compartments. EJ (A) and HCT116 (B) cells that express mCE2, sCE2 or erCE2 were sorted on a FACS for similar eGFP fluorescence. The numbers of parental and mCE2, sCE2 and erCE2 expressing EJ cells (C) and HCT116 cells (D) at the indicated times are shown (n = 3). Bars, s.d.

Cells with similar eGFP intensity were harvested (Fig 4A and 4B). There was no obvious difference in cell growth rate between CE2-expressing and non-expressing cells (Fig 4C and 4D), indicating that CE2 expression did not hinder cell growth.

**Fig 5:**
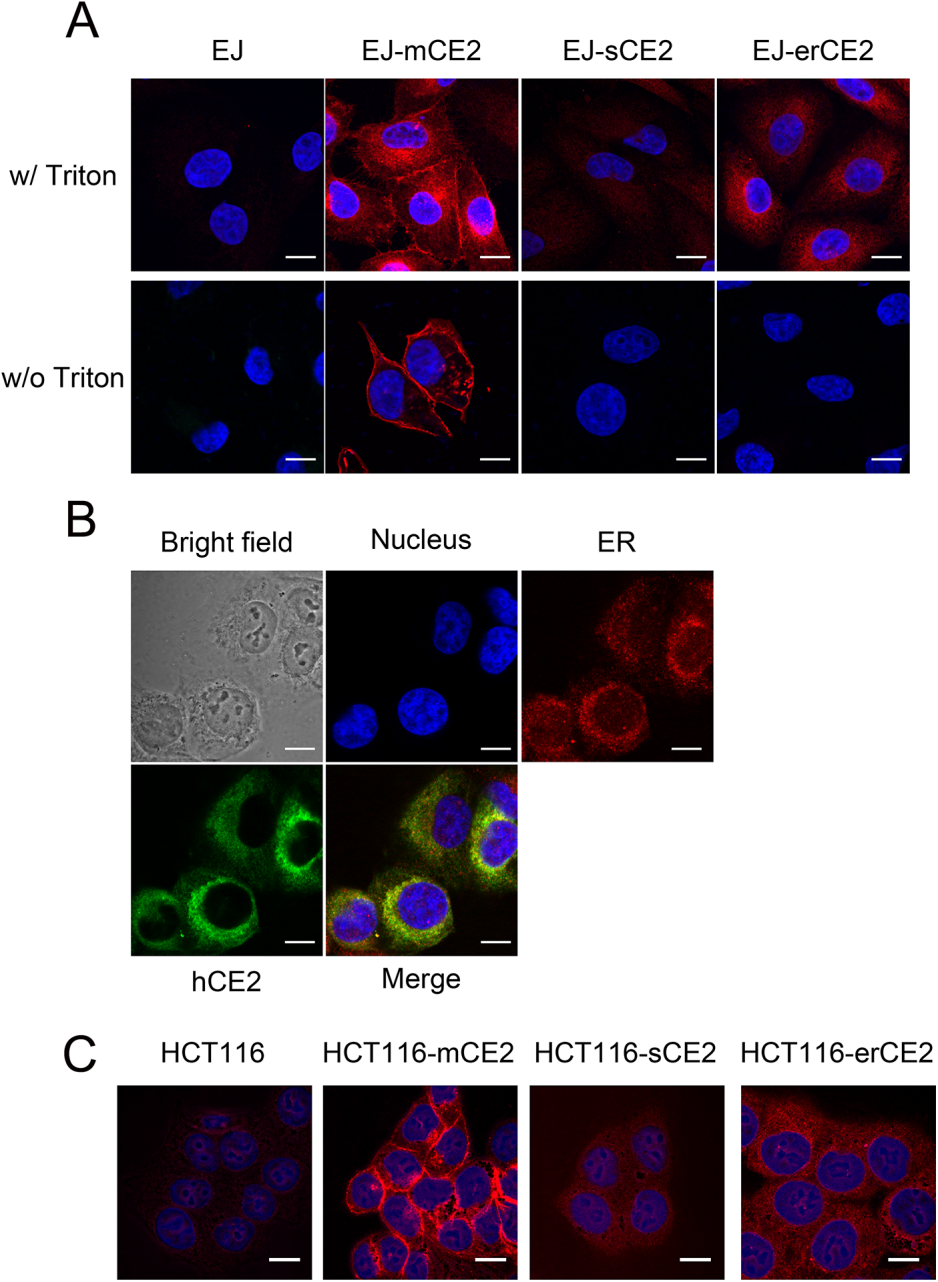
Cellular distribution of CE2. (A) EJ, EJ-mCE2, EJ-sCE2 and EJ-erCE2 cells were treated with PBS (w/o Triton) or Triton X-100 (w/ Triton) to permeabilize the cell membrane. Cells were stained with anti-HA antibody to detect recombinant CE2 location. DAPI indicates the nucleus. (B) EJ-erCE2 cells were triple stained with DAPI (blue), anti-KDEL (red) and anti-HA (green) antibodies to show the distribution of the nucleus, ER and CE2 inside of the cells. (C) HCT116, HCT116-mCE2, HCT116-sCE2, and HCT116-erCE2 cells were pretreated with Triton X-100 and then stained with anti-HA antibody to detect the location of recombinant CE2. Blue indicates the nucleus and red indicates CE2. Scale bar, 10 μm.

Immunofluorescence staining with an anti-HA antibody confirmed the cellular location in different CE2-expressing EJ cells (Fig 5A). EJ-sCE2 cells displayed less fluorescence intensity, corresponding to secretion of CE2 out of the cells. EJ-mCE2 cells not only showed strong fluorescence on the plasma membrane but also inside the cells. Non-permeabilized EJ-mCE2 cells exhibited a ring of fluorescence, confirming that the enzyme was anchored on the plasma membrane. EJ-erCE2 cells displayed a perinuclear localization in permeabilized cells but intact cells were not stained, confirming an intracellular enzyme location. CE2 in EJ-erCE2 cells colocalized with an ER-marker anti-KDEL antibody (Fig 5B), confirming that CE2 in EJ-erCE2 cells was located in the ER. Similar CE2 expression profiles were also observed in HCT116 cells (Fig 5C).

### CE2 protein expression and enzyme activity

**Fig 6:**
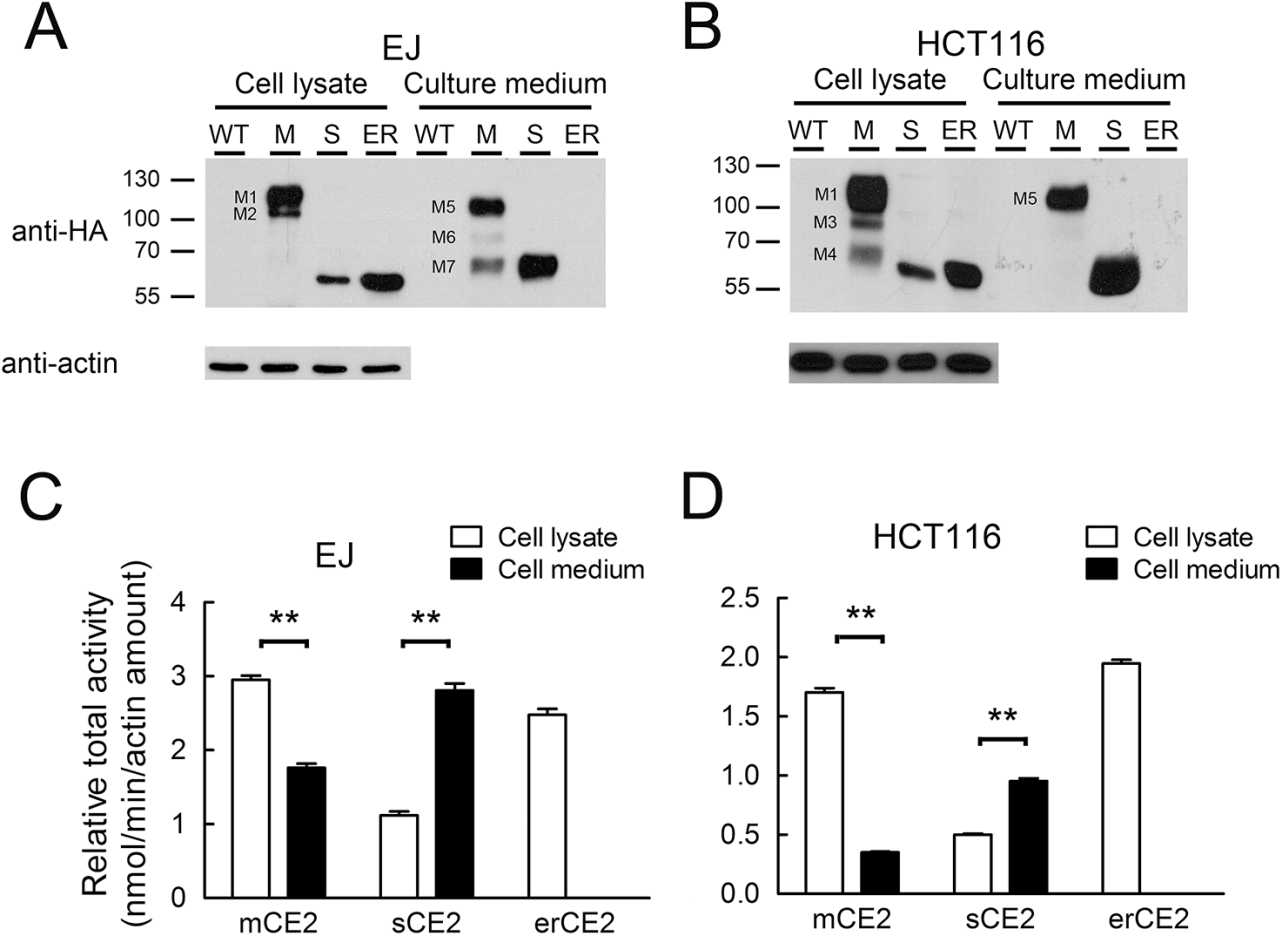
The CE2 expression levels and enzyme activity of different CE2- expressing cells. Western blotting of recombinant CE2 in cell lysates and the culture medium of (A) EJ and (B) HCT116 cells that do not express recombinant CE2 (WT) or express mCE2 (M), sCE2 (S) or erCE2 (ER). Recombinant CE2 was detected by immunoblotting with an anti-HA antibody. M1, M2, M3, and M4 are different CE2 fragments detected in the cell lysate of EJ-mCE2 and HCT16-mCE2 cells. M5, M6 and M7 are different CE2 fragments detected in the culture supernatant of EJ-mCE2 and HCT16-mCE2 cells. Anti-beta-actin staining was used as a cell loading control. The relative specific activity of CE2 in the cell lysate (open bars) or culture medium (closed bars) of EJ cells (C) and HCT116 cells (D) that express the indicated forms of CE2 are shown. n = 3. Bar, s.d. **, P < 0.005

Since the expression of CE2 in different cellular location did not impact the cell growth rate, we seeded the same cell number of parental, mCE2, sCE2, and erCE2 expressing cells, respectively, in the same medium volume. The cell lysate and culture medium were harvested at the same time schedule. Therefore, we can compare the enzyme activity fairly between these cells. Cell lysates and the culture medium prepared from the various EJ cancer cells (Fig 6A) or HCT116 cancer cells (Fig 6B) were immunoblotted with an anti-HA epitope tag antibody to measure CE2 protein levels. Anti-beta-actin antibody was used as a cell loading control. In agreement with the immunofluorescence results, the most CE2 was found in cell lysates prepared from cells expressing mCE2 or erCE2. mCE2 displayed a larger molecular size as compared to sCE2 and erCE2 due to the presence of the membrane-anchoring domain. Large amounts of CE2 were apparent in the culture medium from EJ-sCE2 cells whereas no CE2 could be detected in the culture medium of erCE2 cells. A surprisingly large amount of mCE2 was also detected in the culture medium of mCE2 cells, suggesting that mCE2 could be released from the plasma membrane of the cells.

The cell lysate and culture medium of the cells were incubated with pNPA substrate to measure CE2 enzymatic activities. The enzyme activities were then normalized by the amounts of actin to control for differences in cell numbers. It has been shown that there is a good correlation between the enzyme activity and protein level. EJ-mCE2 and EJ-erCE2 cells had the highest CE2 activities in the cell lysates whereas both EJ-sCE2 and EJ-mCE2 cells had high CE2 activity in the culture medium (Fig 6C). No CE2 enzymatic activity was detected in the culture medium of EJ-erCE2 cells. Similar trends were observed in HCT116 cells (Fig 6D).

### Sensitivity of cells to CPT-11

**Fig 7:**
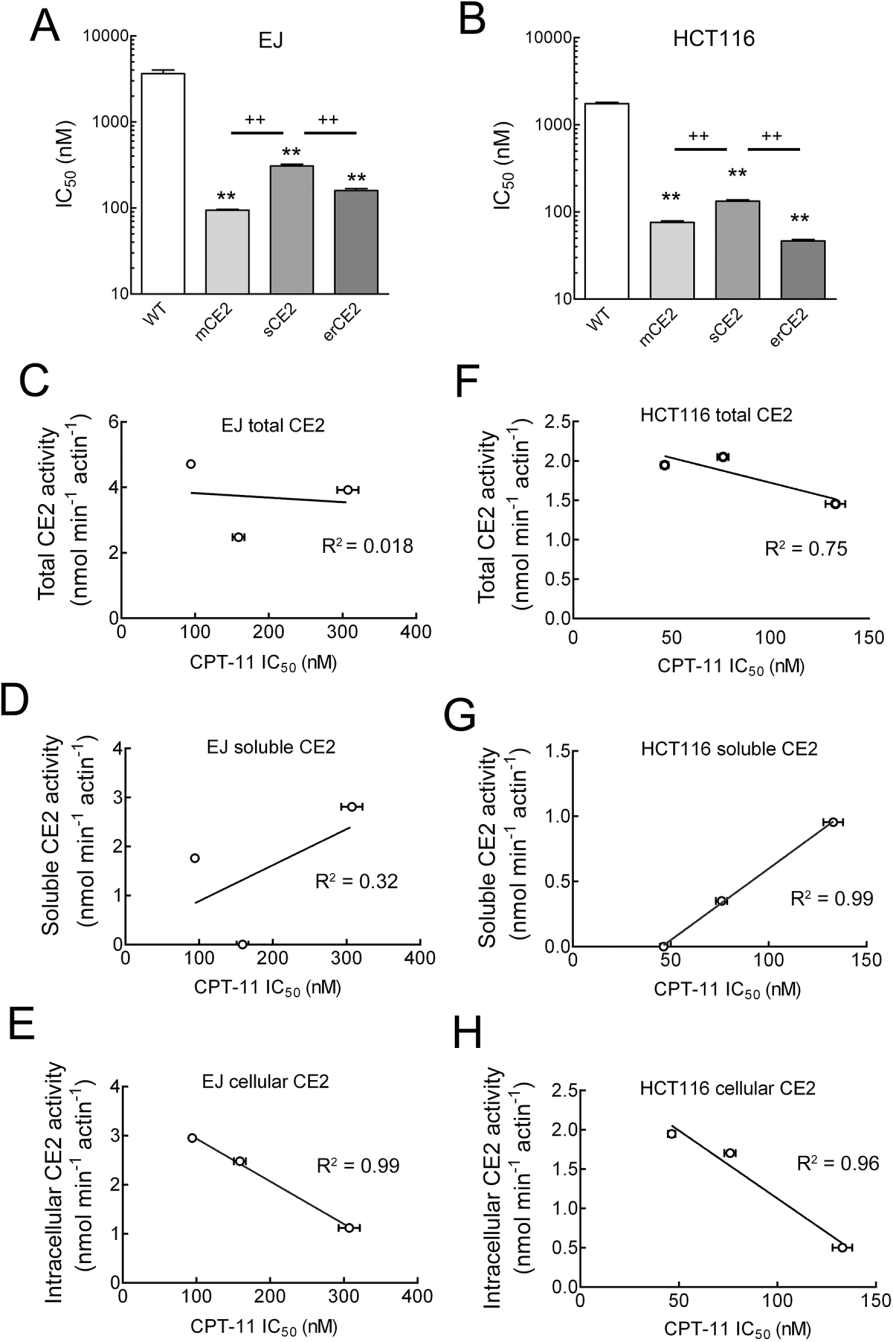
Cellular sensitivities of CE2-expressing cells to CPT-11. (A) EJ cells and (B) HCT116 cells that do not express recombinant CE2 (WT) or express mCE2, sCE2 or erCE2 were incubated with graded concentrations of CPT-11 for 48 h. Cells were incubated with fresh medium for an additional 24 h before incorporation of ^3^H-thymidine incorporation into newly synthesized DNA was measured. Results show the IC_50_ values of CPT-11 to the cells. n = 3. Bars, s.d. Significant difference between CE2-expressing cells compared to WT cells were indicated; ** indicated P < 0.005. Significant difference between CE2-expressing cells compared with the indicated CE2-expressing cells were indicated; ++ indicated P < 0.005. Correlations between cellular sensitivity to CPT-11 and total CE2 activity (soluble plus intracellular) in EJ (C) and HCT116 (F) cells, between cellular sensitivity to CPT-11 and soluble CE2 activity in EJ (D) and HCT116 (G) cells or between cellular sensitivity to CPT-11 and intracellular CE2 activity in EJ (E) and HCT116 (H) cells expressing mCE2, sCE2 or erCE2.

CE2 can convert the prodrug CPT-11 to the topoisomerase I poison SN-38. We therefore determined the sensitivity of the various cancer cells to CPT-11. EJ and HCT116 cells that expressed CE2 were significantly more sensitive to CPT-11 than wild-type cancer cells regardless of the location of the CE2 protein (Fig 7A and 7B). Cells that expressed erCE2 or mCE2 were significantly more sensitive to CPT-11 than cells that expressed sCE2 (Fig 7A and 7B). For EJ cells, IC_50_ values of CPT-11 were lowered by 38, 23 and 12-fold for respectively EJ-mCE2, EJ-erCE2 and EJ-sCE2 cells as compared to parental EJ cells whereas the IC_50_ values were lowered by 23, 38 and 13-fold for respectively HCT116-mCE2, HCT116-erCE2 and HCT116-sCE2 cells as compared to parental HCT116 cells (Table 1). Analysis of the relationship between enzyme activity and IC_50_ values demonstrated a weak correlation between total CE2 activity (both cell-associated and in the culture medium) and cellular sensitivity to CPT-11 (Fig 7C and 7F). By contrast, the activity of soluble CE2 in the culture medium was actually inversely related to cell sensitivity to CPT-11, with higher IC_50_ values observed in cells that displayed the highest soluble CE2 concentrations (Fig 7D and 7G). On the other hand, there was a good correlation (R^2^ = 0.99 and 0.96 for EJ and HCT116 cells, respectively) between cellular CE2 activity and IC_50_ values (Fig 7E and 7H). These results suggest that cell associated CE2 can more effectively enhance CPT-11 antiproliferative capacity as compared to secreted CE2.

**Table 1:** IC_50_ of SN-38 and CPT-11 to different CE2-expressing EJ cells.

Cells	IC_50_ (nM)		QIC_50_
	SN-38	CPT-11	
EJ	4.3 ± 0.17	3600 ± 650	1
EJ-mCE2	2.9 ± 0.06	94 ± 4.0	38
EJ-sCE2	3.5 ± 0.09	310 ± 25	12
EJ-erCE2	4.2 ± 0.14	160 ± 14	23
HCT116	4.3 ± 0.15	1750 ± 108	1
HCT116-mCE2	3.2 ± 0.11	76 ± 5.0	23
HCT116-sCE2	3.4 ± 0.19	133 ± 8.7	13
HCT116-erCE2	3.1 ± 0.18	46 ± 3.3	38

Results show the mean IC_50_ values in nM of triplicate determinations ± standard deviation. QIC_50_ = CPT-11 IC_50_ of EJ cells/CPT-11 IC_50_ of CE-expressing cells.

## Discussion

The cellular location of prodrug-activating enzymes can dramatically influence cellular sensitivity to anticancer prodrugs. Here we examined the effect of expressing human CE2 in three cellular locations on cancer cell sensitivity to CPT-11. We linked eGFP to CE2 expression via a F2A sequence to allow selection of EJ bladder cancer and HCT116 colorectal cancer cells that expressed comparable amounts of ER-retained, secreted or membrane-anchored CE2. All forms of CE2 significantly sensitized the cancer cells to CPT-11, but the membrane-anchored and ER-retained forms of CE2 were more effective than CE2 that was secreted from the cells.

CEs are ubiquitously expressed enzymes which are mainly present in the ER. In 1998, Potter and colleagues observed that the C-terminal HIEL sequence of rCE and hCE1 was crucial for retention of rCE and hCE1 in the ER [18]. To improve the CPT-11 anti-cancer effect, CEs have previously been expressed inside [10, 15–18] or outside [18–23] of cells (Table 2). Although both Potter *et al* [18] and Oosterhoff *et al* [33] generated cells expressing secreted CE or ER-retained CE and showed that secreted CE sensitized cancer cells to CPT-11 more efficiently than ER-retained CE, it is difficult to compare the influence of CE cellular location on CPT-11 therapeutic efficacy due to variations in cell lines examined and differences in gene expression levels between groups with different CE cellular locations. We overcame this problem by linking the expression of CE2 to eGFP via a F2A sequence. The benefits of using a 2A sequence for multiple protein expression include small size (~60–70 bp) and high degree of coordinate expression between target genes [30]. F2A, derived from food-and-mouth disease virus (FMDV), is one of the most commonly used 2A peptides [34, 35]. We found that eGFP fluorescence was strongly correlated with the expression of CE2. Fluorescence-activated cell sorting was used to isolate populations of cells that expressed similar eGFP fluorescence, and by extension, comparable CE2 expression. This allowed us to directly compare the effect of expressing CE2 in different cellular compartments in the same cell lines. This approach represents an easy and convenient method to select cells with tunable protein expression levels.

**Table 2:** Comparison of IC_50_ and QIC_50_ from CPT-11 treated CE-expressing cells.

Author	Cellular location	Cell type		Cell lines	Enzyme species	CPT-11 IC_50_ (nM) ^a^	QIC_50_ ^b^
Khanna ^[^ ^10^ ^]^	ER	Kidney fibroblast		COS-7	hCE2	500	10.8
Yano ^[^ ^15^ ^]^	ER	Malignant lymphomas		U266	hCE2	500	19.6
Potter ^[^ ^16^ ^]^	ER	Kidney fibroblast		COS-7	rCE	920	8.9
		Glioblastoma		U373 MG		78	77
Wierdl ^[^ ^17^ ^]^	ER	Glioblastoma		U373 MG	rCE	400	39
				U373 MG	hCE2	840	18
				U373 MG	hCE1m6	180	86
Oosterhoff ^[^ ^33^ ^]^	ER	Colorectal cancer		SW1398	hCE2	NR ^c^	NR ^c^
	secreted					NR ^c^	NR ^c^
	membrane					NR ^c^	NR ^c^
Porter ^[^ ^18^ ^]^	ER	Kidney fibroblast		COS-7	rCE	950	21
	secreted			COS-7		2400	8.3
Wierdl ^[^ ^23^ ^]^	secreted	Lung carcinoma		A549	rCE	1200	46
		Colorectal Adenocarcinoma		HT29		2100	11
		Neuroblastoma		NB-1691		1200	11
		Rhabdomyosarcoma		Rh30		300	64
		Neuroblastoma		SK-N-AS		2200	14
		Glioblastoma		U373 MG		110	127
Uchino ^[^ ^20^ ^]^	membrane	Gastric cancer		NUGC3	hCE2	NR ^c^	NR ^c^
Danks ^[^ ^22^ ^]^	secreted	Fetal human telencephalon cells		HB1.F3.C1	rCE	NR ^c^	NR ^c^
Oosterhoff ^[^ ^19^ ^]^	secreted	Osteosarcoma		SaOs-2	hCE2	0.7	2857
				MG-63		20	175
				CAL-72		30	83.3
				MNNG-HOS		400	10
				OS-1A		400	7.5
				OS-2		9000	>11.1
				OS-6		30000	>3.3
				OS-6A		1500	>66.6
				OS-7		1100	9
				OS-8		300	66.6

a. IC_50_ is the half maximal inhibitory concentration of CPT-11 for CE-expressing cells.

b. QIC_50_ = IC_50_ of mock cells/IC_50_ of CE-expressing cells.

c. The IC_50_ and QIC_50_ values were not reported.

The cellular location of some prodrug-activating enzymes can greatly alter the effectiveness of anticancer prodrug therapy [36, 37]. For example, beta-glucuronidase that was anchored on the plasma membrane or secreted from target cells was orders of magnitude more effective than intracellularly-expressed beta-glucuronidase for sensitizing cells to glucuronide anticancer prodrugs [38]. Here we found that expression of CE2 on the cell membrane or in the ER sensitized EJ and HCT116 cancer cells to CPT-11 significantly better than did secreted CE2. However, in contrast to beta-glucuronidase, the cellular location of CE2 did not dramatically alter cellular sensitivity to CPT-11. The differences between beta-glucuronidase and CE2 might be attributable to the physical properties of the prodrugs. The glucuronide moiety is charged under physiological pH, which greatly hinders passage of glucuronide prodrugs across the plasma membrane of cells [38]. Thus, glucuronide prodrugs cannot effectively contact beta-glucuronidase unless it is expressed on the cell membrane or is secreted from target cells. By contrast, CPT-11 is relatively lipophilic and appears to be able to penetrate into cells [16]. Thus, even CE2 that is exclusively located intracellularly can effectively sensitize cancer cells to CPT-11.

We found significant amounts of CE2 in the cell lysate and culture medium of cells that expressed membrane-anchored CE2 (Fig 6A and 6B). These bands are likely caused by differences in glycosylation and protease degradation. Human CE2 possesses two N-linked glycosylation sites whereas the extracellular domain of the B7 tether possesses an additional three N-linked glycosylation sites [39, 40]. The major M1 band, corresponding to the full length, completely glycosylated membrane-tethered CE2, was present in the cell lysate of both EJ and HCT116 cells. Smaller fragments present in the cell lysate (M2, M3 and M4) most likely represent proteolytic cleavage products. Differences in the ER quality control and the spectrum of proteases present in EJ versus HCT116 cells may cause a different range of proteolytic products, some of which appear to be retained inside the cells [41]. Based on the molecular weights of the bands and knowledge that the HA epitope tag used for immunoblotting is present at the N-terminus of CE2, the M2 and M3 fragments likely resulted from cleavage within the extracellular domain of B7. Cleavage near the myc tag may have generated the M4 fragment. Soluble CE2 was also present in the culture medium of EJ-mCE2 and EJ-erCE2 cells. The major soluble CE2 band (M5) was slightly smaller than membrane-anchored CE2, suggesting cleavage of the B7 tether near the cell membrane. We have observed similar release of other membrane-anchored proteins into the culture medium including alpha fetoprotein [42], single-chain antibodies [43] and beta-glucuronidase [26]. Alterations in the glycosylation, degradation and retention of mCE2 in EJ and HCT116 cells may be responsible for the differences in CE2 fragments in the culture medium of EJ-mCE2 and EJ-erCE2 cells.

Although sCE2-expressing cells displayed high CE2 activity in the culture medium, soluble CE2 did not appear to efficiently sensitize cancer cells to CPT-11 (Fig 7D and 7G). On the other hand, we observed a good correlation between cell-associated CE2 activity and cellular sensitivity to CPT-11 (Fig 7E and 7H). These results suggest that SN-38 generated inside the cells or near the cell membrane might display greater activity as compared to SN-38 generated outside the cells in the culture medium. It is well known that camptothecin and its derivatives can strongly interact with plasma proteins such as human serum albumin (HSA), which can dramatically decrease their anticancer activity [42–45]. Likewise, conjugation of HSA with SN-38 increased the solubility of SN-38 but also decreased its cytotoxicity to cancer cells by over 10 fold [46]. Binding of SN-38 to serum proteins likely reduces the rate of cell uptake. Thus, SN-38 generated by hydrolysis of CPT-11 by soluble CE2 in the culture medium may be partially neutralized by binding to serum proteins. By contrast, hydrolysis of CPT-11 by intracellular or membrane-tethered CE2 may reduce SN-38 neutralization by serum proteins, allowing more effective inhibition of topoisomerase I. Expression of CE2 in cells may also help avoid potential off target prodrug activation, recommending this cellular compartment for enhancement of CPT-11 anticancer activity.

Besides the cellular location of CE2, other factors, such as genetic polymorphisms and the expression levels of enzymes involved in CPT-11 metabolism, can also affect the therapeutic efficacy of CPT-11 treatment. For example, the activity of topoisomerase I, the cellular target of SN-38, has been correlated with cellular sensitivity to CPT-11 [44, 45]. SN-38 can also be metabolized to a glucuronide conjugate in the liver by uridine diphosphate glucuronyltransferase (UDPGT) [46]. UDPGT genetic polymorphisms can therefore affect the toxicity of CPT-11 via alternation of SN-38 bioavailability in patients [47, 48]. Over expression of the MRP multidrug resistance protein is also involved in resistance to CPT-11 and SN-38 [49].

In summary, we found that CE2 can sensitize cancer cells to CPT-11 regardless of whether the enzyme is expressed inside or outside the cells. However, expression of CE2 in the ER or on the plasma membrane of cancer cells more effectively sensitized cancer cells to CPT-11 as compared to secreted CE2. We suggest that expression of CE2 in the ER of target cells may allow efficient activation of CPT-11 without the danger of systemic prodrug activation.
